# Fungal Carbonic Anhydrases: A Systematic Review from Molecular Profiling to Pathogenic Regulation in *Magnaporthe oryzae*

**DOI:** 10.3390/jof12080555

**Published:** 2026-07-26

**Authors:** Yujia Li, Yanxia She, Tingzhen Wang, Yutong Liu, Shuyuan Wang, Songhang Hu, Cong Liu, Yuejia Dang

**Affiliations:** 1 School of Life and Health, Dalian University, Dalian 116622, China; liyujia@s.dlu.edu.cn (Y.L.); sheyanxia@s.dlu.edu.cn (Y.S.); wangtingzhen@s.dlu.edu.cn (T.W.); liuyutong@s.dlu.edu.cn (Y.L.); wangshuyuan@s.dlu.edu.cn (S.W.); husonghang@s.dlu.edu.cn (S.H.); liucong@s.dlu.edu.cn (C.L.); 2 Institute of Modern Agriculture Research, Dalian University, Dalian 116622, China

**Keywords:** carbonic anhydrase, fungi, *Magnaporthe oryzae*, pH homeostasis, pathogenicity, mitochondrial function

## Abstract

Carbonic anhydrases (CAs) are a class of zinc-containing metalloenzymes widely present in the biological world, catalyzing the reversible hydration of CO_2_ to form HCO_3_^−^ and H^+^. These enzymes play essential roles in pH homeostasis, gas exchange, metabolic regulation, and virulence expression in pathogens. In fungi, CAs mainly belong to the α- and β-classes and have undergone extensive diversification during evolution. In plant pathogenic fungi, the functions of CAs have extended beyond traditional metabolic roles, evolving into key “environmental adaptation and virulence regulatory factors.” This review takes *Magnaporthe oryzae* as a model organism and integrates recent advances in CA research across various microorganisms. It systematically summarizes the classification diversity, structural features, subcellular localization, and biological functions of fungal CAs. Particular emphasis is placed on the molecular profile, mitochondrial localization, physical interaction network, and multiple functional roles of the MoCA family members in conidial development, appressorium formation, oxidative stress response, HCO_3_^−^ homeostasis, nitrogen metabolism, and mitochondrial energy metabolism. Based on these findings, this study proposes a multi-layered analytical framework integrating CA molecular characteristics, mitochondrial functional regulation, and fungal pathogenicity. It explores the potential of targeting fungal CAs for the development of novel selective fungicides and highlights key research directions, aiming to provide theoretical insights into plant-fungal interactions and innovative strategies for disease control.

## 1. Introduction

Carbonic anhydrase (CA, EC 4.2.1.1) is a widely distributed metalloenzyme found in various organisms, including animals, plants, bacteria, and fungi. Its primary function is to catalyze the reversible reaction between carbon dioxide (CO_2_) and water (H_2_O), producing bicarbonate (HCO_3_^−^) and a proton (H^+^) [1]. This reaction plays a crucial role in physiological and pathological processes such as pH regulation, gas exchange, and metabolic activities [2,3], and is also involved in the growth and virulence of pathogens, including bacteria, fungi, and protozoa [4]. In fungi, particularly in plant pathogenic species like *Magnaporthe oryzae*, CAs are increasingly recognized for their roles in responding to environmental changes and physiological demands, thereby influencing fungal growth, development, and interactions with hosts [5,6].

In recent years, significant progress has been made in understanding the role of CAs in human pathogenic fungi [7]. For instance, in *Candida albicans*, the β-CA acts as a CO_2_ scavenger, playing a critical role in pathogenicity under CO_2_-limited environments such as epithelial surfaces [8,9]. In *Cryptococcus neoformans*, two β-CA genes, *CAN1* and *CAN2*, are involved in CO_2_ sensing and virulence regulation during infection of human hosts [10]. Similarly, in *Malassezia* spp., β-CA has been identified as a key virulence factor [11,12]. These findings highlight the essential role of fungal CAs in cell survival, proliferation, and CO_2_ signaling cascades associated with pathogenicity and differentiation.

In contrast, research on CAs in plant pathogenic fungi has lagged behind. While plant CAs have been shown to play roles in host resistance, the involvement of pathogen CAs in disease progression remains largely unexplored. To address this gap, our team conducted the first functional characterization of β-CA (MoCA1) in *M. oryzae*. Subsequent studies have further explored other members of the MoCA family, including MoCA2, MoCA4, MoCA5, and MoCA6, gradually revealing the significance of CAs in plant-pathogen interactions. To comprehensively elucidate the multifaceted roles of fungal CAs in plant-pathogen interactions, *M. oryzae* was selected as the central model organism. As one of the most destructive rice pathogens globally, *M. oryzae* poses a serious threat to global food security. Its infection cycle—comprising conidia germination, appressorium formation, penetration peg development, and hyphal expansion—is well characterized and amenable to genetic manipulation. Additionally, the *M. oryzae* genome contains at least six CA-encoding genes, providing a solid foundation for functional studies. Our group has accumulated extensive data on the MoCA family members (MoCAs), making this system ideal for in-depth investigation. By thoroughly analyzing the role of MoCAs in *M. oryzae* and integrating findings from other microbial systems, this review not only reveals unique mechanisms but also provides a paradigm for understanding the conservation and diversity of CAs in pathogenic microorganisms.

## 2. Fungal Carbonic Anhydrases Present a Molecular Landscape Shaped by Both Their Characteristic Features and Associated Regulatory Mechanisms

### 2.1. Diversity of the Fungal CA Family

The carbonic anhydrase (CA) family is composed of eight distinct evolutionary branches: α, β, γ, δ, π, η, θ, and ι. Among these, α-CA is the most abundant class, and is widely distributed across animals, higher plants, algae, protozoa, fungi, and bacteria [13,14,15]. In contrast, β-CA is found in all organisms except mammals [6,14,16,17,18,19]. Based on amino acid sequence and structural features, fungal CAs are primarily classified into α- and β-type classes [4].

In fungi, the majority of CAs belong to the β-class, while α-CA is predominantly found in filamentous Ascomycetes [6,8,20]. The CA-encoding genes in fungi have undergone extensive diversification during evolution. Most filamentous Ascomycetes contain three β-CA isoenzymes and at least one α-CA, whereas in hemiascomycetous yeasts, only β-CA has been identified so far [8]. For example, *Saccharomyces cerevisiae* [21], *C. albicans* [22], and *Candida glabrata* [7] each encode a single β-CA, while the pathogenic basidiomycete *C. neoformans* contains two β-CA genes [15,23]. Some filamentous Ascomycetes, such as *Sordaria macrospora*, possess three β-CA homologs (CAS1, CAS2, and CAS3) [8].

According to the EnsemblFungi database (http://fungi.ensembl.org/index.html), Uniprot (https://www.uniprot.org/) and recent studies, the rice blast fungus *M. oryzae* (strain 70-15) encodes at least six CA family members, which have been named MoCA1 through MoCA6. Our team conducted a systematic classification of these genes. Among them, MoCA1 (*MGG_04611*) was the first functionally characterized β-CA with significant Zn^2+^-dependent CO_2_ hydration activity [24], while MoCA4 (*MGG_06600*), MoCA6 (*MGG_09234*), and MoCA5 (*MGG_18017*) are also β-CAs [25,26]. MoCA2 (*MGG_04973*) and MoCA3 (*MGG_01692*) are α-CAs [25]. All these MoCAs consist of an N-terminal arm, a zinc-binding core region, and a C-terminal subdomain.

**Figure 1:**
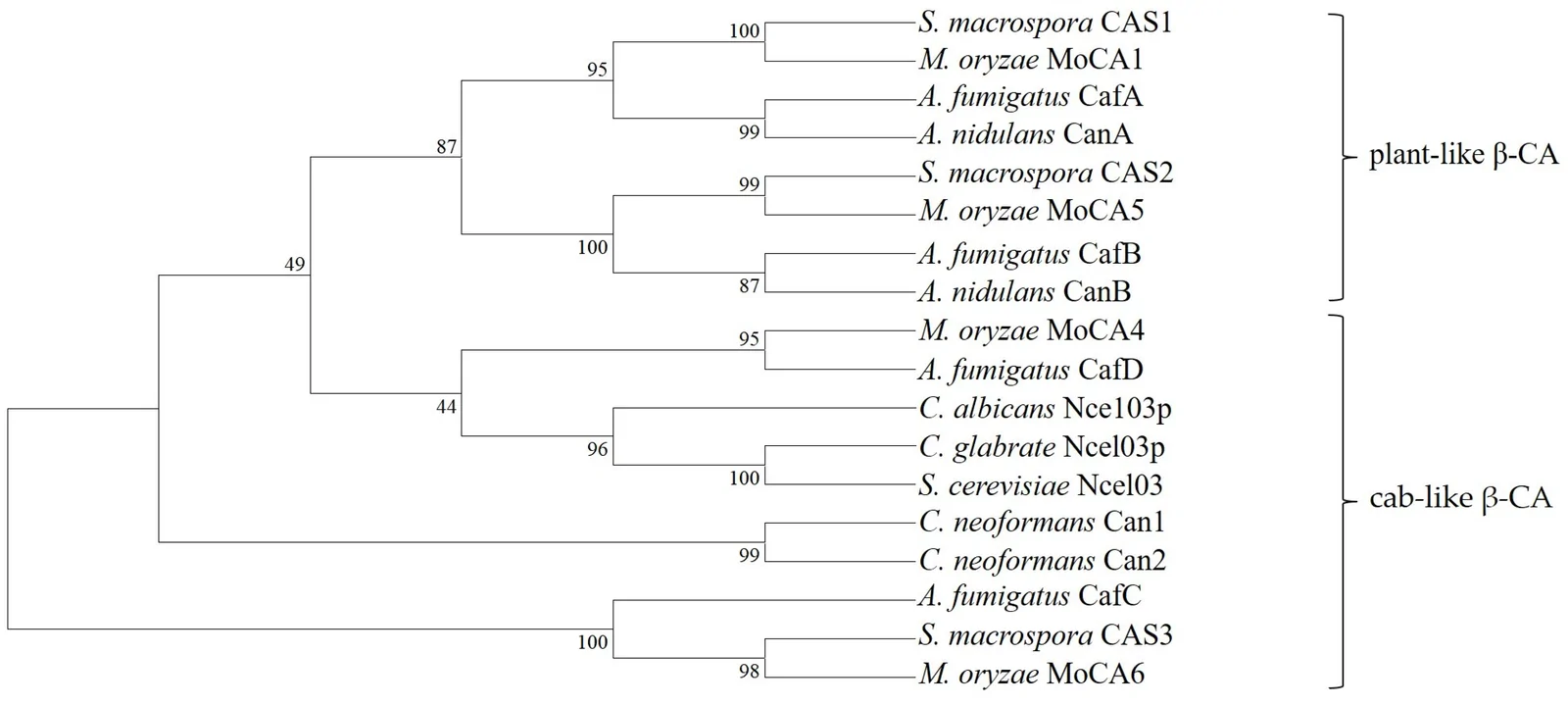
A phylogenetic tree of fungal β-CAs is presented, with the types of β-CAs indicated on the right side. All sequences were obtained from the NCBI databases (https://www.ncbi.nlm.nih.gov/).

Phylogenetic analysis reveals that β-CA can be further divided into plant-like β-CA, cab-like β-CA, and ε-like β-CA [27,28]. For instance, *Aspergillus fumigatus* contains four β-CA genes (*cafA*–*cafD*), while *Aspergillus nidulans* has two β-CA genes (*canA* and *canB*), which belong to different evolutionary clades: CafA/CanA belongs to the plant-like β-CA1 subclade (Clade I), CafB/CanB belongs to the plant-like β-CA2 subclade (Clade II), and CafC/CafD belongs to the cab-like β-CA3 subclade (Clade III/IV) [29]. In *S. macrospora*, the three β-CA homologs include CAS1 and CAS2, which belong to the plant-like CA subfamily, and CAS3, which is a distantly related cab-type enzyme [8]. As shown in Figure 1, we compiled the phylogenetic tree of most fungal β-CAs and classified the *M. oryzae* β-CAs. MoCA1 is closely related to CAS1 and belongs to the plant-like β-CA1 subclade (Clade I); MoCA5 is closely related to CAS2 and belongs to the plant-like β-CA2 subclade (Clade II); MoCA4 is closely related to CafD and belongs to the cab-like β-CA3 subclade (Clade III/IV); and MoCA6 is closely related to CAS3 and belongs to a distantly related cab-type enzyme. Many filamentous Ascomycetes possess one gene encoding a cab-type β-CA and two genes encoding plant-like β-CAs, but some species encode multiple cab-type CAs, suggesting that these two ancestral genes may have undergone multiple gene duplication events during fungal evolution. In-depth studies of β-CAs in ancient fungal lineages may help reveal the origin and evolutionary trajectory of this gene family [8].

Based on the phylogenetic branching, it is evident that although these enzymes originate from the same species as β-CAs, their amino acid sequence variations at key residues within the active sites lead to distinct structural configurations of the hydrophobic pocket. This divergence is robustly supported by evolutionary analysis, which clearly resolves them into two separate clades. In plant-like β-CA, the hydrophobic pocket is constituted by three conserved residues, namely Gln151, Phe179, and Tyr205, which together define a characteristic hydrophobic microenvironment. In contrast, the corresponding residues in the cab-like β-CA are not conserved, and exhibit variability across different cab-type enzymes, allowing for substitutions at these positions [8,30].

Overall, CAs in fungi have undergone extensive diversification during evolution and perform diverse physiological functions in different species. These enzymes are not only essential for fungal growth and survival but also play important roles in their interactions with the environment.

### 2.2. Structural Features of Fungal CAs

The CA family is evolutionarily independent, showing low sequence similarity but highly conserved active site structures [31]. All CAs contain a mononuclear metal-binding site, which plays an indispensable role in enzyme performance: a polarized/ionized metal-bound water molecule acts as a nucleophile, attacking incoming CO_2_ [1]. Notably, despite the lack of significant sequence homology among different CA classes, the catalytic centers of α-, β-, δ-, and η-CAs all depend on zinc ions (Zn^2+^) as a key cofactor [4]. Zn^2+^ coordinates with three amino acid residues and a water/hydroxide molecule, forming a tetrahedral configuration that activates the water molecule and facilitates the hydration of CO_2_. This “structurally diverse yet functionally conserved” feature makes CAs ideal subjects for studying enzymatic catalytic mechanisms and evolutionary biology [32].

**Figure 2:**
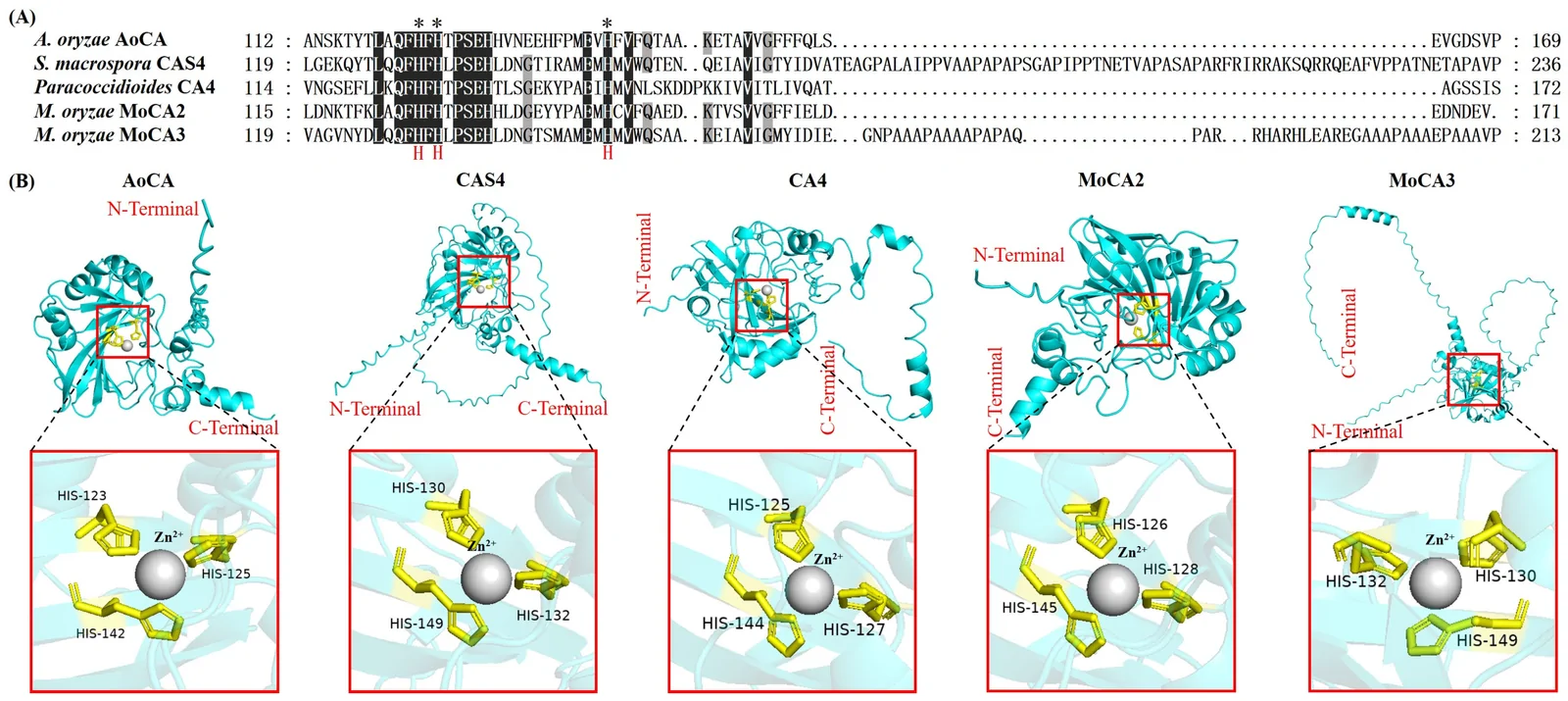
Schematic diagrams of α-CAs sequences and structures. (**A**) Multiple sequence alignment of representative α-CAs from *A. oryzae*, *S. macrospora*, *Paracoccidioides*, and *M. oryzae*. Conserved amino acids important for Zn^2+^ coordination are marked with asterisks. Identical conserved amino acids across all proteins are shown in black shading; residues conserved in at least four sequences are shown in dark gray shading. (**B**) Three-dimensional structural images of representative α-CAs from *A. oryzae*, *S. macrospora*, *Paracoccidioides*, and *M. oryzae*. Zinc ions are shown as gray spheres. Catalytic histidines are labeled in yellow with specific sites indicated, and the N-Terminal and C-Terminal are annotated with labels.

Known bacterial and fungal α-CAs typically exist as dimers, whereas most mammalian α-CAs are monomers, with some existing as dimers [33,34,35]. The active site of fungal α-CA contains a zinc ion coordinated by three conserved histidine residues and a water molecule, following a two-step CO_2_ hydration mechanism [36]. Among fungal α-carbonic anhydrases (α-CAs), *Aspergillus oryzae* α-CA (AoCA) is the only one whose structure has been determined at the atomic level. AoCA exists as a homodimer, with each monomer composed of eight central β-strands and six α-helices. The active site contains three histidine residues (His 123, His 125, and His 142) that coordinate a zinc ion, while Q121, E140, and N249 help stabilize the spatial orientation of the zinc ion. Notably, the classical proton shuttle residue H64 is replaced by phenylalanine F99 in this enzyme. Additionally, AoCA contains an intramolecular disulfide bond between Cys58 and Cys219, and it is glycosylated at 11 sites [18,36]. In contrast, the structure of *S. macrospora* CAS4 is primarily inferred through sequence analysis and predicted to be a secreted glycoprotein. Its active site includes three conserved histidine residues (His 130, His 132, and His 149) involved in catalysis, along with stabilizing residues (Q123, E149, and N332). The position corresponding to the proton shuttle in other α-CAs is occupied by threonine instead of histidine. This protein also retains cysteine residues that may form disulfide bonds [36,37]. The *Paracoccidioides* CA4, when expressed recombinantly, forms a monomeric secreted protein consisting of 301 amino acid residues. Its sequence contains a conserved zinc-binding motif [36,38]. In *M. oryzae*, two α-carbonic anhydrases, MoCA2 and MoCA3, have been identified. Their active sites contain three conserved histidine residues: His126, His128, and His145 for MoCA2 [25], and His130, His132, and His149 for MoCA3. These residues are highly conserved compared to those in other α-CAs (Figure 2).

**Figure 3:**
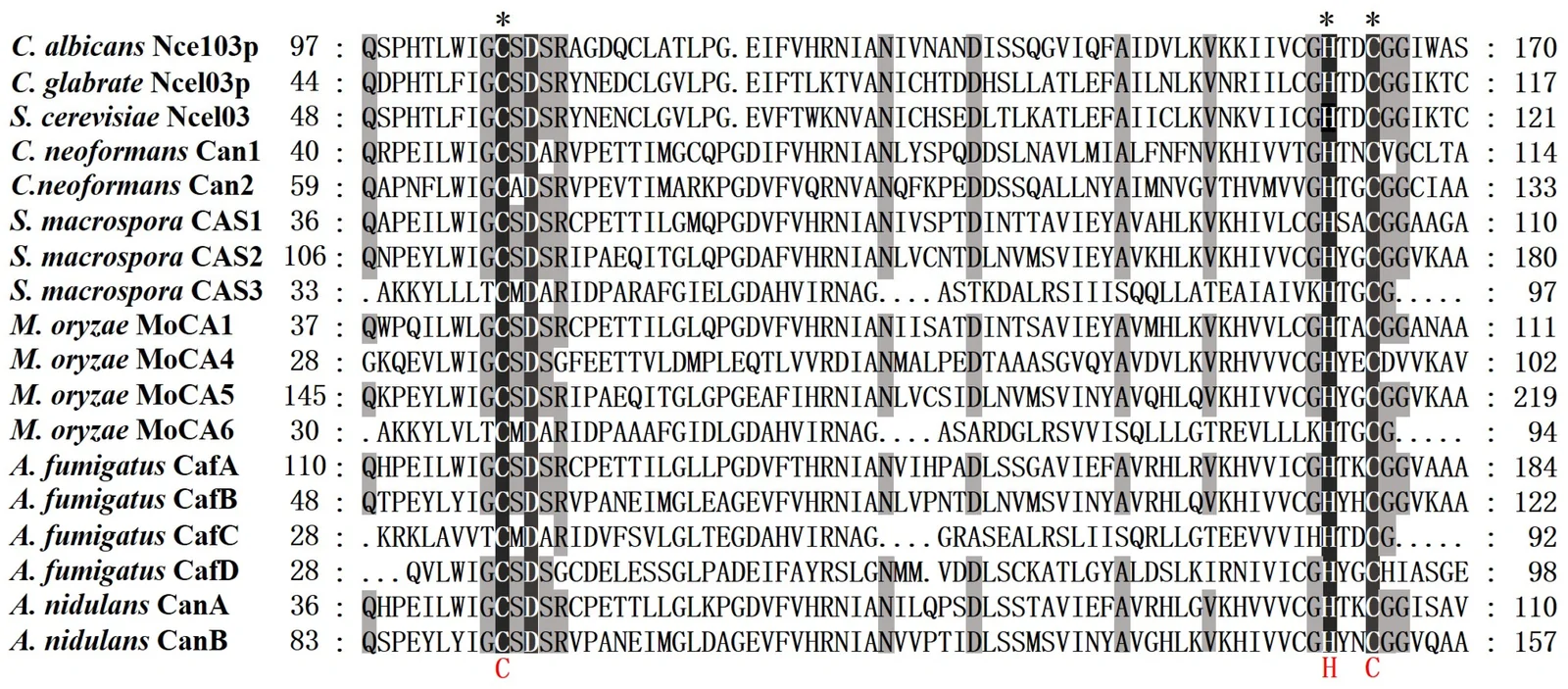
Multiple sequence alignment of fungal β-CAs. Conserved amino acids important for Zn^2+^ coordination are marked with asterisks. Identical conserved amino acids across all proteins are shown in black shading; residues conserved in at least 13 sequences are shown in dark gray shading. All sequences were obtained from the NCBI databases (https://www.ncbi.nlm.nih.gov/). Multialignment analyses were conducted using the MEGA 12.0 and GeneDoc 2.7.000 software packages.

**Figure 4:**
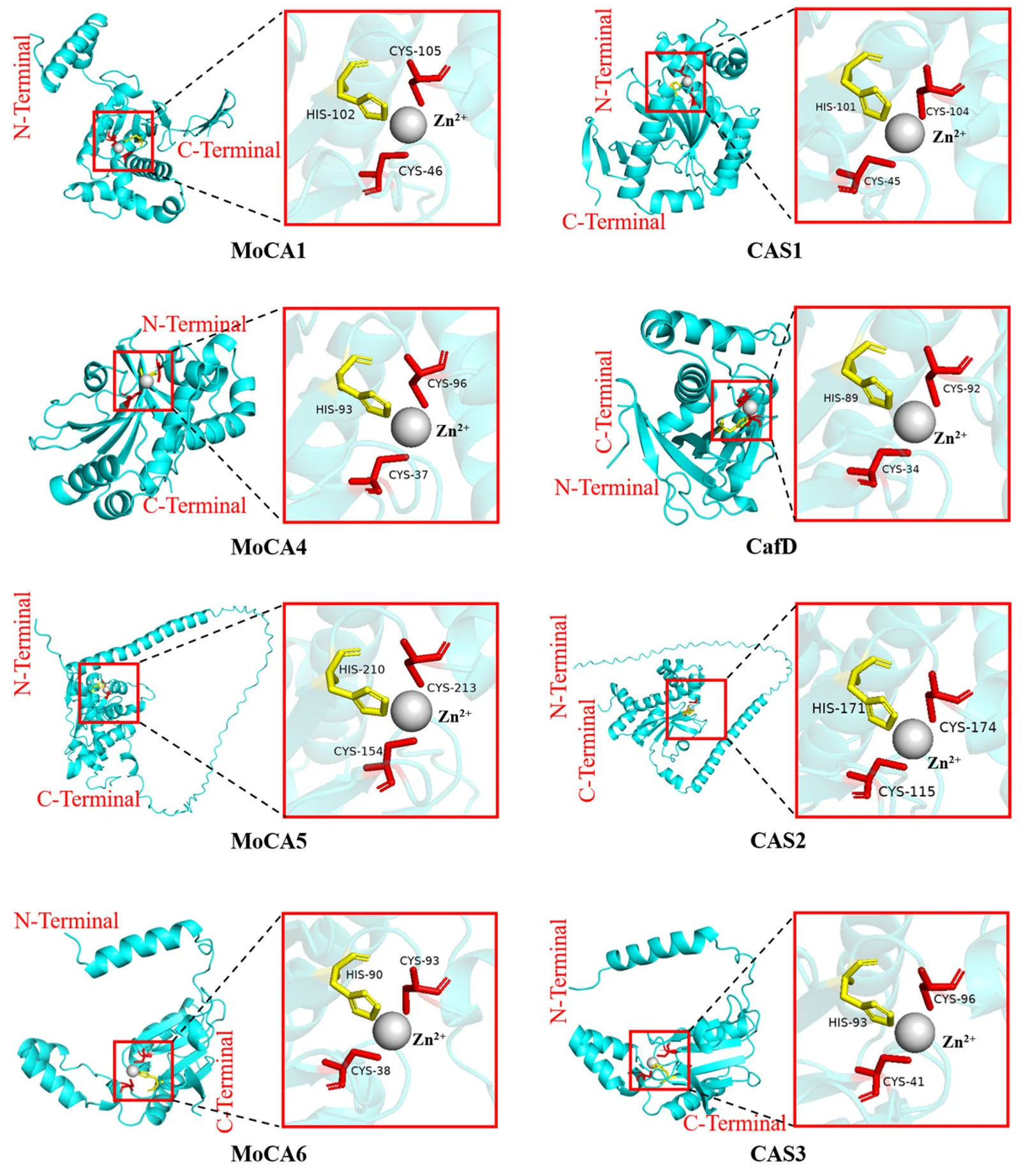
Three-dimensional structural images of representative β-CAs. Zinc ions are shown as gray spheres. Catalytic cysteines are labeled in red with specific sites indicated, catalytic histidines are labeled in yellow with specific sites indicated, and the N-Terminal and C-Terminal are annotated with labels.

Fungal β-CAs form oligomeric structures ranging from dimers to octamers [4,39], such as plant-like tetramers and cab-like dimers [32]. The zinc ion in these enzymes is coordinated by two cysteine and one histidine residue. The protein sequences and structures of most fungal β-CAs are shown in Figure 3 and Figure 4. For instance, the active site of *S. cerevisiae* Nce103 is located at the interface between two monomers, with the zinc ion coordinated by three highly conserved residues: Cys57, His112, and Cys115 [40]. *A. fumigatus* CafA is a special β-CA whose high-resolution crystal structure reveals a tetramer formed by dimeric dimers, with the catalytic zinc ion coordinated by three conserved residues (Cys119, His175, and Cys178) and an acetate anion, exhibiting an “open” conformation [32]. In contrast, CafD adopts a unique “closed” conformation, in which the zinc ion is directly coordinated by four amino acid residues—Cys34, His89, Cys92, and Asp36—with aspartate Asp36 replacing the typical water molecule as the fourth ligand, thereby locking the enzyme in an inactive state. Furthermore, CafD contains an additional non-catalytic zinc-binding site at the dimer interface, mediated by residue Cys39; this structural zinc ion contributes to the stabilization of its distinctive dimeric assembly, a feature that is absent in CafA, CafB, and CafC [41]. In *S. macrospora*, in addition to the two Cys and one His residues that coordinate the Zn^2+^, CAS1, CAS2, and CAS3 each possess one Asp and one Arg residue that are highly conserved in structure. These residues are implicated in proton shuttling, substrate binding, or serving as the fourth zinc ligand. Although all three enzymes share the same core catalytic mechanism, CAS2 differs from the other two by possessing an N-terminal extension that encodes a mitochondrial targeting sequence [42]. In *M. oryzae*, the zinc-binding core of MoCA1, MoCA4, MoCA5, and MoCA6 consists of two cysteine and one histidine residue [24,25,26]. Additionally, structural differences among CA types reflect adaptive strategies of organisms to their environments and provide important models for studying protein folding and function relationships [43].

### 2.3. Physical Interactions and Functional Synergy Among CA Family Members

There are significant physical interactions and functional synergies among MoCA family members. Our team confirmed via yeast two-hybrid (Y2H) and bimolecular fluorescence complementation (BiFC) assays that MoCA1 interacts physically with MoCA2, MoCA4, MoCA6, and MoCA5 in mitochondria [25,26]. Functionally, MoCA family members except for MoCA3 exhibit compensatory expression regulation. For example, the expression levels of other members were significantly upregulated in the Δ*MoCA6* mutant, suggesting that MoCA6 may act as a “hub” within the family. Furthermore, CA inhibitor-acetazolamide (Ace, 50 nM) treatment experiments showed that multiple *MoCAs* knockout mutants were highly sensitive to Ace, and Ace could suppress the expression of *MoCAs* genes in the wild-type strain of *M. oryzae*, further revealing the synergy and dependency among MoCA family members [25,26].

### 2.4. Expression Regulation Serves as a Critical Link Between Environmental Signals and Carbonic Anhydrase Function

The expression of CAs is precisely regulated by various environmental signals, forming the molecular basis for their role as “environmental sensors.” Studies have shown that MoCA1 expression in *M. oryzae* exhibits significant developmental stage specificity, being notably upregulated during conidiophore development and early infection stages. Additionally, environmental stressors such as H_2_O_2_ and NaHCO_3_ significantly induce its expression, indicating that MoCA1 not only participates in normal fungal development but also responds to external environmental changes, playing a role in stress adaptation [24]. Similarly, in *C. albicans* and *C. neoformans*, CA expression is regulated by CO_2_ concentration, with the bZIP transcription factor Rca1p identified as a key regulatory factor. This regulatory mechanism enables CAs to sense and respond to changes in external CO_2_ levels, thereby modulating cellular physiology and adaptive behaviors [25]. In summary, the expression regulation of CAs is closely associated with environmental signals in different fungi, serving as a crucial bridge connecting external environmental stimuli with the execution of CA functions. It highlights the central role of CAs in fungal adaptation to environmental changes and maintenance of physiological homeostasis.

## 3. Subcellular Localization and Functional Differentiation of Fungal Carbonic Anhydrases

### 3.1. Diversity in Subcellular Localization

**Figure 5:**
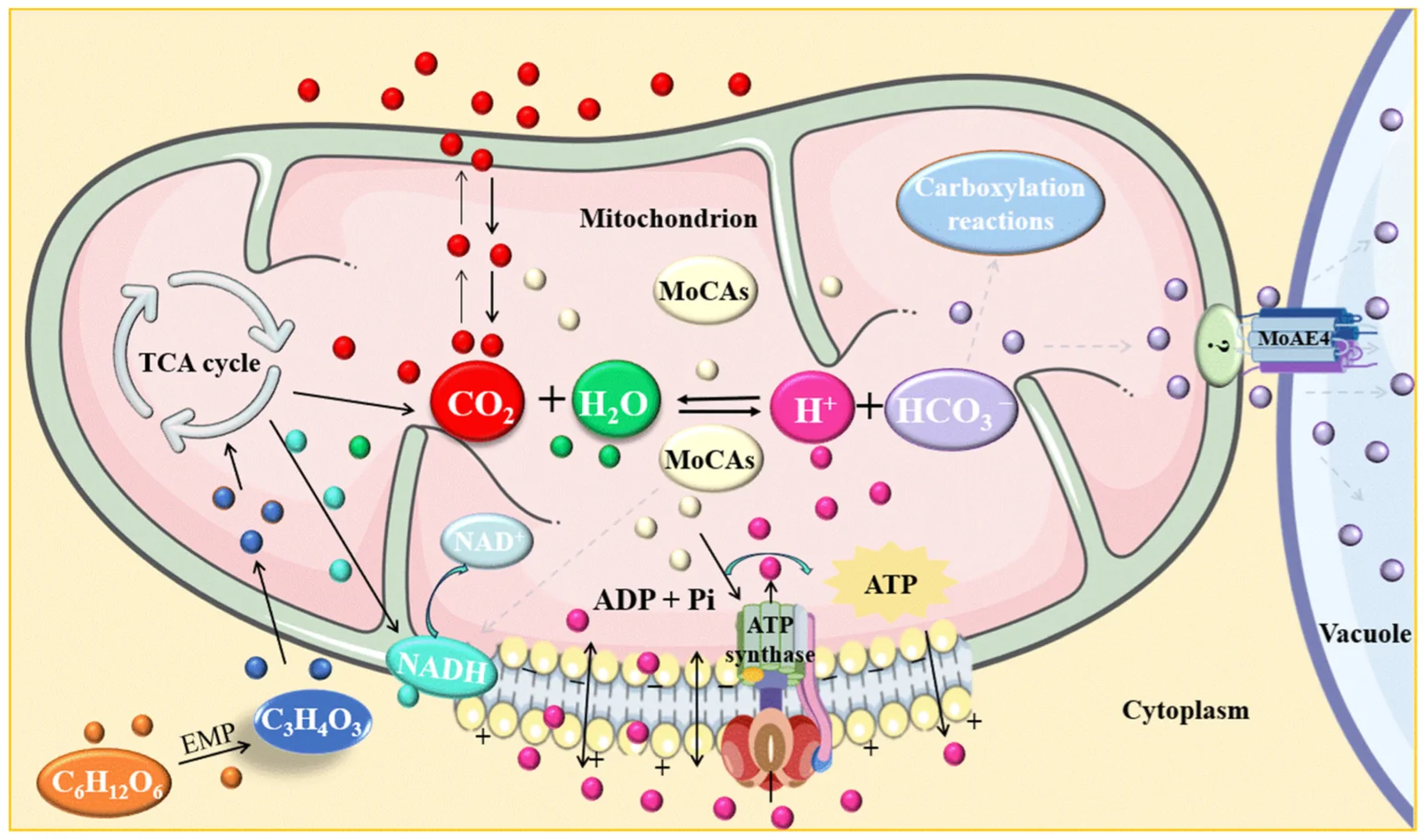
A schematic model depicting the functional mechanism of carbonic anhydrases in *M. oryzae* during the process of plant infection The ATP and mitochondria models were created using BioGDP.com [44] CO_2_ hydration module: MoCAs catalyze CO_2_ hydration to HCO_3_^−^ and H^+^, maintaining mitochondrial inorganic carbon balance and matrix pH homeostasis. Energy coupling module: MoCA-generated H^+^ contributes to proton homeostasis and cooperates with respiratory complexes to sustain membrane potential and ATP production. Carbon/nitrogen metabolic coupling module: Glycolysis (EMP)-derived pyruvate enters mitochondria and fuels the TCA cycle, generating CO_2_. CA catalyzes the conversion of CO_2_ into HCO_3_^−^, supplying substrates for HCO_3_^−^-dependent carboxylation reactions. Color codes: red, CO_2_; green, H_2_O; pink, H^+^; purple, HCO_3_^−^; cyan, NADH; light blue, NAD^+^; yellow, MoCAs; orange, C_6_H_12_O_6_; dark blue, C_3_H_4_O_3_.

Fungal carbonic anhydrases (CAs) are localized to various subcellular compartments, including the cytoplasm, mitochondria, secretory, and membrane-associated isoforms. This localization determines their functional specificity and reflects functional differentiation [8]. The subcellular localization of CAs in different fungal species is summarized in Table 1. Studies have shown that Nce103p CA in *C. albicans* and *C. parapsilosis* is primarily localized to the cell wall and plasma membrane, but it also partially resides in the cytoplasm and mitochondria. Its localization is speculated to be related to its role in CO_2_ sensing and regulation of intracellular bicarbonate (HCO_3_^−^) levels [9]. In *S. macrospora*, CAS2 is localized in the mitochondria, while CAS1 and CAS3 are localized in the cytoplasm, indicating some functional differences between them [6]. In *A. fumigatus*, CafA is predicted to be a mitochondrial protein, which undergoes processing by cleavage of its N-terminal mitochondrial targeting sequence to form the mature form [43]. However, studies on the subcellular localization of five members of the carbonic anhydrase (CA) family in *M. oryzae* (MoCA1, MoCA2, MoCA4, MoCA5, and MoCA6) have shown that all these proteins are localized to the mitochondria, as demonstrated by fluorescent protein tagging experiments [24,25,26]. Further Y2H and BiFC experiments confirmed that MoCA1 physically interacts with MoCA2, MoCA4, MoCA5, and MoCA6 within the mitochondria [25,26], further supporting the reliability of the mitochondrial localization of these proteins. Based on these findings, it can be inferred that *M. oryzae* may concentrate multiple CA family members within the mitochondria to form a mitochondria-localized CA functional network, which collaboratively regulates mitochondrial-related functions. This mitochondrial aggregation may reflect a unique evolutionary strategy employed by plant pathogenic fungi to meet high energy demands and adapt to metabolic changes during infection. As the central hub of cellular energy metabolism, the mitochondria play a critical role in the infection process of pathogenic fungi. This mechanism also highlights the metabolic regulation strategies adopted by pathogens in response to host immune defenses, as illustrated in Figure 5.

### 3.2. Functional Differentiation of Carbonic Anhydrases

To date, research on α-CAs remains relatively limited, only four fungal α-CAs have been identified, including those from *A. oryzae*, *S. macrospora*, *Paracoccidioides*, and *M. oryzae*. The functions of α-CAs in different fungal species are summarized in Table 1. Among these, the α-CA from *A. oryzae*, primarily exhibits catalytic function, capable of reversibly catalyzing the hydration of carbon dioxide. However, its catalytic efficiency is relatively low due to the substitution of the classical proton shuttle residue with phenylalanine (F99) in the active site. This reduced activity can be restored by exogenous imidazole [36]. The CAS4 from *S. macrospora* is a secreted α-CA that plays a critical role in fungal physiology, particularly in nutritional growth and ascospore germination. It facilitates the utilization of carbon sources under atmospheric CO_2_ conditions by catalyzing the hydration of CO_2_ to produce bicarbonate. The absence of this enzyme leads to a significant decrease in growth rate and a shift toward aquatic growth to seek a more CO_2_-rich microenvironment [36,37]. In *Paracoccidioides*, the α-CA CA4 is closely associated with fungal pathogenicity and is considered a potential virulence factor. Its expression is significantly upregulated during liver infection in the host, and it is speculated to provide essential bicarbonate for the synthesis of malonyl-CoA, thereby participating in fatty acid biosynthesis. This function may help the fungus adapt metabolically and survive within the host [36,38]. Additionally, MoCA2 from *M. oryzae* plays a crucial role in conidia formation, growth, development, and pathogenicity, and is involved in the regulation of mitochondrial function, ATP synthesis, and nitrogen metabolism via the glutamine–glutamate pathway [25]. These studies highlight the potential roles of fungal α-CAs in metabolic adaptation, physiological regulation, and pathogenic mechanisms. However, further investigation is needed to fully elucidate their molecular mechanisms and biological significance.

In fungi, particularly the widely present β-class CAs, carbonic anhydrases play an essential role in growth, differentiation, survival, and virulence by catalyzing the reversible conversion between CO_2_ and HCO_3_^−^ [32]. The functions of β-CAs in different fungal species are also summarized in Table 1. In fungi such as *C. albicans*, *S. cerevisiae*, *C. neoformans*, *S. macrospora*, *A. nidulans*, and *A. fumigatus*, CA-mediated HCO_3_^−^ synthesis is a necessary condition for normal growth in ambient air [7]. Research has shown that the α-type carbonic anhydrase in *S. cerevisiae* is essential under ambient air conditions, and its transcription is regulated by inorganic carbon concentration [8]. In pathogenic yeasts such as *C. albicans*, *A. fumigatus* and *C. neoformans*, CA genes are also essential under ambient air conditions. For example, the *CAN2* gene in *C. neoformans* can fully restore the CO_2_-dependent phenotype in *S. cerevisiae* when expressed heterologously [8,9]. In terms of CO_2_ sensing and signal transduction, CA is a core component of the fungal CO_2_ sensing system. For instance, *CAN2* in *C. neoformans* has been proven to be critical for CO_2_ sensing [10]. In *C. albicans* and *C. neoformans*, HCO_3_^−^ generated by CA directly activates adenylyl cyclase, triggering the cAMP-PKA signaling pathway [6]. *C. albicans* requires carbonic anhydrase to adapt to pH changes in host tissues and survive under hypoxic or high CO_2_ conditions. Studies have shown that CA gene knockout mutants of *C. albicans* exhibit significantly reduced virulence in both in vitro and in vivo models, confirming that pathogenic fungi rely on carbonic anhydrase to adapt to the host environment and establish infection [4].

In sexual reproduction and conidia formation, CA is essential for completing the sexual reproductive cycle in various filamentous ascomycetes (such as *S. macrospora*) and yeasts (such as *C. neoformans*) [39,45]. Multiple β-CAs (CAS1, CAS2, CAS3) and one α-CA (CAS4) in *S. macrospora* participate in fruiting body formation and ascospore germination, with overlapping yet differentiated functions. Specifically, CAS2 (mitochondrial localization) is crucial for hyphal growth and conidia germination, and its absence leads to severe developmental delay [6,8]. In *C. neoformans*, Can2 generates HCO_3_^−^ to activate adenylyl cyclase, increasing intracellular cAMP levels and thereby regulating mating and spore formation. The Δ*can2* mutant fails to complete conidia formation due to insufficient HCO_3_^−^ [46]. In *A. fumigatus*, *CAs* (*cafA*, *cafC*) genes affect conidial production, indicating their involvement in conidia development regulation [27]. In *A. nidulans*, CanA plays an independent role in conidiation [27]. In *M. oryzae*, β-CAs (MoCA1, MoCA4, MoCA5, MoCA6) also influence conidia growth and development, participating in conidia formation, germination, appressorium generation, and even showing that the appressorium malformation rate in knockout mutants is significantly increased, severely affecting conidia development [24,25,26].

In terms of virulence and pathogenicity, for many human pathogenic fungi (such as *C. albicans*, *C. neoformans*, and *A. fumigatus*), CA is essential for establishing infection and expressing virulence in microhabitats (such as the surface of host epithelial cells, where CO_2_ is limited). Knockout of *CA* genes significantly weakens their pathogenic ability in both in vitro and in vivo models. In plant pathogens, β-CAs of *M. oryzae* also participate in the pathogenic process [4,24,25,26].

In terms of participation in metabolic pathways, carbonic anhydrases provide metabolic substrates, and the HCO_3_^−^ generated by their catalysis is a key substrate for various carboxylation reactions, including fatty acid synthesis (e.g., acetyl-CoA carboxylase), amino acid synthesis, and purine synthesis [7]. The CA activity in *S. cerevisiae* and *C. neoformans* is critical for fatty acid synthesis, and the absence of CA results in growth defects under low CO_2_ conditions, which can be partially restored by exogenous palmitic acid, indicating that CA deficiency affects fatty acid synthesis catalyzed by acetyl-CoA carboxylase. Other carboxylation reactions (such as pyruvate carboxylase, aminoimidazole carboxylase) are also affected, but fatty acid synthesis is the main limiting factor [6,45]. In *S. macrospora*, CA provides HCO_3_^−^ for cyanase (CYN1) to degrade cyanate, possibly participating in the regulation of arginine synthesis [6]. In *M. oryzae*, β-CAs of *M. oryzae* also coordinate mitochondrial function, ATP synthesis, and glutamine–glutamate-mediated nitrogen metabolism, thus maintaining cellular pH homeostasis [25,26].

In summary, CAs play a central role in various biological processes, including environmental adaptation, sexual reproduction, spore formation, pathogenicity, and metabolic regulation. By catalyzing the reversible conversion of CO_2_ to HCO_3_^−^, CAs not only maintain intracellular pH homeostasis and inorganic carbon acquisition but also function as signaling molecules that activate the cAMP-PKA pathway, thereby regulating morphological transitions, virulence expression, and stress responses. In human pathogenic fungi such as *C. albicans* and *C. neoformans*, CAs are critical for adapting to the host microenvironment and establishing infection. In contrast, in plant pathogenic fungi like *M. oryzae*, CAs are more involved in mitochondrial function maintenance, energy metabolism, and the formation of infection structures. Although the fundamental catalytic mechanism of CAs is highly conserved, their downstream functional networks exhibit significant specificity depending on the host ecological niche, reflecting the adaptive evolution of fungi to different host environments.

## 4. Core Functional Analysis Reveals the Multiple Roles of MoCAs Throughout the Life Cycle and Pathogenicity of *M. oryzae*

In *M. oryzae*, MoCA family members have evolved into key “environmental adaptation and virulence regulatory factors.” Among them, MoCA3 remains without visible experimental evidence in current studies, while the other members have demonstrated significant functional roles. The following section systematically elaborates on their core functions in the pathogenic cycle from six dimensions.

### 4.1. The MoCAs-MoAE4 Collaborative Pathogenic Model Governs HCO_3_^−^/pH Homeostasis and Ion Balance in M. oryzae

During infection, *M. oryzae* is subjected to various environmental stresses, such as nitrogen limitation, increased HCO_3_^−^ concentration, and hypoxia [47,48,49]. These challenges require robust mechanisms to maintain intracellular pH and metabolic stability. Our research highlights the critical role of the carbonic anhydrase family in regulating intracellular pH homeostasis. These findings provide valuable insights into the molecular strategies used by plant pathogens to adapt to hostile host environments and may inform the development of new disease control methods.

A novel bicarbonate transporter, MoAE4, has been identified in *M. oryzae*, localized to both the plasma and vacuolar membranes. Together with MoCA1, it forms a collaborative pathogenic model that helps maintain HCO_3_^−^/pH balance. Under normal conditions, CO_2_ diffuses freely, maintaining equilibrium with low expression of MoCA1 and MoAE4. However, during early infection, the pathogen encounters a hypoxic, high-CO_2_ environment, leading to cytoplasmic acidification and metabolic disruption [50].

Five MoCA family members are mitochondrial-localized and interact physically, suggesting mitochondria as the central hub for HCO_3_^−^ regulation [24,25,26]. In the infection process of *M. oryzae*, members of the MoCAs family are proposed to catalyze the hydration of CO_2_, generating HCO_3_^−^ and H^+^, thereby maintaining intracellular pH homeostasis and alleviating CO_2_ toxicity. Concurrently, MoAE4, as an HCO_3_^−^ transporter, is capable of transporting excess HCO_3_^−^ from the cytoplasm into the vacuole for storage or secreting it into the extracellular space of plant cells, thus preventing the toxic accumulation of HCO_3_^−^. It is hypothesized that MoCAs and MoAE4 may function in a coupled manner. The HCO_3_^−^ produced by MoCAs can be actively transported by MoAE4, forming a closed-loop regulatory system that participates in the metabolic adaptation and pathogenicity of the pathogen within the host microenvironment. This represents the first reported functional coupling between carbonic anhydrases (CAs) and HCO_3_^−^ transporters in fungi, offering new insights into the adaptive mechanisms employed by fungi under host stress conditions. The hypothetical model presented in Figure 5 further illustrates the coordinated action of MoCAs and MoAE4 during infection, highlighting their critical roles in fungal growth, development, and pathogenicity.

### 4.2. Regulators of Development and Differentiation

The reaction catalyzed by CA directly produces or consumes H^+^, making it one of the fastest regulators of intracellular and extracellular pH. HCO_3_^−^ itself can act as a signaling molecule, and changes in its concentration can be sensed by adenylyl cyclase, thereby modulating cAMP levels and influencing fungal development and pathogenic processes [22,45].

#### 4.2.1. Gatekeeper of Conidial Development

Five MoCA family members play a key regulatory role in conidiophore development and conidia production in *M. oryzae* [24,25,26]. Mechanistically, Dang et al. [24] speculated that *MoCAs* gene deletion may lead to excessive accumulation of CO_2_, disrupting acid-base homeostasis, as a certain concentration of HCO_3_^−^ is essential for efficient sporulation and meiosis [42]. Under normal physiological conditions, MoCAs catalyze the hydration of CO_2_ to generate HCO_3_^−^, maintaining an appropriate intracellular HCO_3_^−^ concentration. When *MoCAs* function is lost, CO_2_ cannot be effectively converted to HCO_3_^−^, leading to CO_2_ accumulation and HCO_3_^−^ deficiency, which disrupts intracellular pH balance and metabolic environment, ultimately inhibiting conidiophore development and conidia production. Cross-species comparisons show that this mechanism also exists in *S. macrospora* and *C. neoformans*, indicating that CA-regulated sporulation is highly conserved among fungi [42,46].

#### 4.2.2. Appressorium Development and Turgor Pressure Generation

The appressorium is a critical structure for *M. oryzae* infection, requiring melanin deposition and turgor pressure as high as 8.0 MPa [51]. Melanin is not only a pigment component of appressoria but also crucial for maintaining cell wall mechanical strength. Its reduction weakens the ability of appressoria to withstand high turgor pressure, leading to penetration failure [52]. Five MoCA mutants exhibit reduced melanin content, increased malformed appressoria, and impaired turgor generation. MoCAs contribute to melanin synthesis by maintaining appropriate HCO_3_^−^ and pH levels, and they also support turgor by providing protons for H^+^-ATPase activity. These functions are conserved across fungal species, highlighting the importance of CAs in appressorial function. Additionally, turgor pressure generation depends on the rapid accumulation of solutes (such as glycerol), a process that requires the continuous pumping of H^+^ by the plasma membrane H^+^-ATPase to establish a proton gradient [53]. MoCAs, by catalyzing the hydration of CO_2_ to produce H^+^ and HCO_3_^−^, may provide a local source of protons for the H^+^-ATPase and help maintain the transmembrane proton gradient. Therefore, *MoCAs* loss may result in insufficient solute accumulation and reduced turgor pressure. This mechanism is conserved across multiple fungi, indicating that CA plays a central role in appressorium development and functional regulation.

#### 4.2.3. Virulence

Three studies consistently confirm that five *MoCAs* deletion leads to a significant decrease in the virulence of *M. oryzae*, including severely impaired conidia infection of leaves and extension of invasive hyphae within host cells [24,25,26]. Mechanistically, the decline in virulence caused by *MoCAs* loss results from multiple defects: reduced sporulation directly decreases the number of infection sources; malformed appressoria and reduced melanin lead to decreased penetration ability; and impaired extension of invasive hyphae reflects the continuous need for MoCAs to maintain intracellular pH homeostasis and energy supply during the biotrophic stage. Cui et al. [25] also showed that there is physical interaction and functional compensation among CA family members (e.g., *MoCA6* upregulation in multiple mutant backgrounds), which may partially compensate for the functional loss caused by single *MoCAs* deletion. However, the integrity of the *MoCAs* network is essential for virulence. Overall, MoCAs comprehensively affect the pathogenic capacity of *M. oryzae* by regulating multiple steps from sporulation to invasive hyphae extension.

### 4.3. MoCAs Act as Key Responders to Environmental Stress, Particularly Oxidative Stress

MoCAs play a critical role in the stress response of *M. oryzae*, particularly under oxidative stress conditions. Dang et al. [24] showed that Δ*MoCA1* mutants accumulate higher levels of H_2_O_2_ compared to wild-type strains, both in rice sheath cells and within hyphae. MoCA1 expression is upregulated in response to H_2_O_2_ and NaHCO_3_, indicating its involvement in both oxidative and pH stress responses [24]. Cui et al. [25] and Dang et al. [] further confirmed that other MoCA members (e.g., MoCA2, MoCA4, MoCA5, MoCA6) also led to increased sensitivity to the CA-specific inhibitor acetazolamide and cordycepin, suggesting that the entire MoCA family members participate in stress adaptation [25,26].

From a mechanistic perspective, it can be inferred that MoCAs influence multiple aspects of the stress response. The loss of MoCAs leads to reduced ATP levels and downregulation of ATPase gene expression, which impairs antioxidant systems such as the glutathione and thioredoxin pathways. Additionally, disruption of the CO_2_/HCO_3_^−^ buffering system affects intracellular pH regulation, reducing metabolic efficiency. As five members of the MoCA family are localized to mitochondria, where they play a role in maintaining matrix pH, dysfunction of these proteins may result in pH imbalance, decreased electron transport efficiency, and increased production of reactive oxygen species (ROS), thereby exacerbating oxidative stress. A slight shift in mitochondrial matrix pH can significantly affect the conformation and activity of the electron transport chain (especially complex I) [54]. MoCAs dysfunction may lead to imbalanced matrix pH, causing a decrease in electron transfer efficiency, increased electron leakage, and subsequent generation of superoxide radicals (O_2_•^−^), ultimately leading to excessive ROS accumulation. This mechanism aligns closely with the discovery of a “carbonic anhydrase domain” in the mitochondrial respiratory chain complex I of plants [54], supporting MoCAs as a central hub connecting pH homeostasis, energy metabolism, and oxidative stress defense in *M. oryzae*’s pathogenic process.

### 4.4. Systematic Regulation of Nitrogen Metabolism

In recent years, the functional research of CAs has made significant breakthroughs, extending beyond traditional pH regulation. They have been confirmed to be closely related to the nitrogen metabolism pathway. Cui et al. [25] and Dang et al. [26] showed that five Δ*MoCAs* mutants exhibit severe growth defects under nitrogen-free conditions, which can be partially rescued by adding glutamine or glutamate. In contrast, no significant growth differences were observed between wild-type and mutant strains in nitrogen-rich media. Molecular-level analysis further revealed that multiple nitrogen metabolism-related genes (e.g., nitrate reductase *MGG_06062*, glutamine synthetase *MGG_06888*/MoGln2, glutamate synthase *MGG_08074*/MoGlt1) were significantly downregulated in Δ*MoCAs* mutants [25,26]. Dang et al. [26] constructed a complete nitrogen metabolism regulatory network model in a cordycepin-treated transcriptome analysis, in which MoCA1 and MoCA5 participated in nitrogen metabolism and were significantly inhibited by cordycepin [26].

Mechanistically, the virulence of plant pathogenic fungi is regulated by multiple cellular pathways that respond to changes in the host environment. Nitrogen limitation has been identified as a key signal triggering the expression of virulence genes in the host [55]. Fungi are often exposed to nitrogen-limited environments, and effective regulation of nitrogen metabolism is crucial for their survival, growth, development, and pathogenicity [56]. MoCAs play a central role in responding to nitrogen source limitations, balancing carbon-nitrogen metabolism, and maintaining metabolic homeostasis during the infection process of *M. oryzae*. Particularly during the infection of plant pathogenic fungi, MoCAs may help them more efficiently utilize limited nitrogen sources through participation in the glutamine–glutamate metabolism, thereby enhancing their pathogenicity. Nevertheless, the molecular intermediaries linking MoCAs to these nitrogen metabolism-related genes remain highly unclear, and there is currently no direct evidence that classic nitrogen-responsive transcription factors play a dominant regulatory role. Moreover, most related phenotypes have not been ruled out as secondary metabolic stress responses rather than specific pathogenic activation. In this context, MoCAs have been proposed to act as a “hub node” for the coordinated regulation of carbon–nitrogen metabolism due to their roles in pH homeostasis, CO_2_/HCO_3_^−^ conversion, and potential metabolite-sensing functions. Whether MoCAs may optimize the redistribution and reuse efficiency of limited nitrogen sources by modulating local HCO_3_^−^/pH microenvironments, thereby affecting the activity or subcellular localization of key enzymes in the glutamine–glutamate cycle (Gln–Glu cycle), also requires further validation. In summary, while the importance of MoCAs as candidate factors linking environmental nitrogen signals to pathogenic metabolic reprogramming is beginning to emerge, current understanding remains deeply entrenched in a “phenotype–association–speculation” cycle of reasoning. Future research urgently needs to overcome three major bottlenecks: (i) developing in situ metabolic imaging techniques suitable for living infected tissues to analyze the spatiotemporal dynamics of MoCA activity; (ii) constructing conditional knockout/active site mutants combined with targeted metabolomics (e.g., ^15^N-labeled nitrogen flux tracing) to rigorously test their quantitative contribution to Gln–Glu cycle flux; and (iii) integrating yeast two-hybrid, Co-IP-MS, and proximity labeling (BioID/APEX) techniques to systematically map the in vivo interaction network of MoCAs, with particular attention to their potential cross-kingdom interactions with mitochondrial nitrogen metabolism enzyme complexes and host-derived nitrogen transporters. Through these multi-dimensional, high-precision research strategies, it is expected that the core role of MoCAs in the metabolic pathogenesis of the rice blast fungus will be truly revealed, providing new theoretical foundations and research directions for understanding the metabolic regulatory mechanisms by which pathogenic fungi respond to environmental stress.

### 4.5. Carbonic Anhydrases Play an Energetic Role in Supporting Mitochondrial Function and ATP Synthesis

Mitochondrial membrane potential is a key indicator of mitochondrial functional integrity and the driving force for ATP synthesis [57]. Cui et al. [25] and Dang et al. [26] showed that five MoCAs mutants exhibit reduced mitochondrial membrane potential and significantly lower ATP levels compared to the wild type. The expression of key ATP synthase subunit genes, such as α-subunit, β-subunit, M4, and M9, is also downregulated, highlighting the role of MoCAs in mitochondrial energy production [25,26].

Based on findings in plant research, such as the presence of a “carbonic anhydrase domain” in the mitochondrial respiratory chain complex I of Arabidopsis thaliana, which may provide a local source of H^+^ for proton transport [58], it is speculated that MoCAs may have a similar function: catalyzing the hydration of CO_2_ to generate H^+^, thereby providing a local source of protons for proton translocation by complex I, which in turn enhances proton pumping efficiency. Studies have shown that HCO_3_^−^ not only serves as a substrate for bicarbonate-dependent enzymes such as pyruvate carboxylase, but also contributes to the replenishment of oxaloacetate (OAA) and anaplerotic reactions in the tricarboxylic acid (TCA) cycle [59]. Moreover, HCO_3_^−^ may also act as a substrate for carbamoyl phosphate synthetase, participating in the biosynthesis of arginine and pyrimidine nucleotides [30]. Therefore, it is proposed that MoCAs may regulate the pH of the mitochondrial matrix, maintaining the optimal catalytic environment for ATP synthase, and modulating HCO_3_^−^ homeostasis to influence related metabolic reactions, thereby regulating the coupling between the TCA cycle and oxidative phosphorylation (OXPHOS) (as shown in Figure 5). In addition, the loss of MoCAs may lead to the downregulation of ATP synthase subunit gene expression, suggesting that MoCAs may be involved in the regulation of mitochondrial gene expression.

Since the pathogenic process of *M. oryzae* is highly dependent on ATP-driven processes such as appressorium turgor formation, invasive hyphal expansion, and effector secretion [60,61,62], MoCAs may support these energy-intensive processes by maintaining mitochondrial function and ATP synthesis capacity. Therefore, the impairment of ATP synthesis caused by MoCAs deletion may be a key factor in the reduction in virulence.

In summary, MoCAs has been demonstrated to be a critical node in the energy metabolism of *M. oryzae*, with its physiological significance confirmed through genetic experiments. However, the mechanistic understanding of MoCAs remains largely based on heuristic analogies from plant models, lacking specific biochemical, structural, and dynamic metabolic evidence in fungi. Future research must overcome three major challenges: (1) the development of mitochondria-targeted pH/HCO_3_^−^ nanoprobes suitable for live hyphae, enabling subcellular-scale dynamic monitoring; (2) the elucidation of the ultrastructural localization of MoCAs within the mitochondria and its interaction network, to determine whether it forms functional complexes; and (3) the construction of conditionally and reversibly controllable MoCAs activity inhibition systems (such as chemical genetics tools), to circumvent developmental compensation effects and precisely dissect the energy regulatory roles of MoCAs at different stages of infection. Only through such efforts can we move beyond descriptive observations and truly reveal how carbonic anhydrase has evolved from an ancient “metabolic cofactor” into a key energy hub for the pathogenic adaptation of *M. oryzae*.

## 5. Applications of Fungal Carbonic Anhydrases

### 5.1. Antifungal Drug Development

Clinically, antimicrobial agents commonly inhibit microbial growth by interfering with protein and nucleic acid synthesis, cell wall biosynthesis, membrane permeability, or essential metabolic pathways [63]. However, the current arsenal of antifungal drugs remains limited, and the emergence of drug resistance is increasingly threatening clinical efficacy—making the development of novel therapeutics targeting previously unexploited molecular vulnerabilities an urgent priority. Genome-wide comparative analyses between pathogenic and non-pathogenic microbes have identified CAs as promising candidate targets for antimicrobial intervention [64]. Rational design of CA inhibitors (CAIs) thus offers a compelling strategy to develop next-generation antifungals with novel mechanisms of action.

CAs catalyze the reversible interconversion of CO_2_ and HCO_3_^−^, playing indispensable roles in pH homeostasis, carbon supply, and core metabolic processes across diverse microorganisms. Pharmacological inhibition of CA activity disrupts this finely tuned equilibrium, leading to impaired metabolism, defective stress adaptation, and attenuated virulence [49,58]. Indeed, CAIs are emerging as broad-spectrum anti-infective agents: classical inhibitors such as ethoxzolamide demonstrate potent in vitro bactericidal activity against *Helicobacter pylori*, a major gastric pathogen [64]. In dermatomycology, the lipophilic yeasts *M. globosa* and *M. restricta*—key etiological agents of dandruff and seborrheic dermatitis—are susceptible to sulfonamides, sulfamates, and KI-containing sulfonamide derivatives, which induce hyphal fragmentation and growth arrest [11,12].

Notably, β-class CAs—structurally distinct from mammalian α-CAs but widely conserved and functionally essential in fungi—represent an ideal target for selective antifungal development. Simple inorganic anions (e.g., cyanate, thiocyanate) and metal-complex anions—well-established classes of CAIs—effectively inhibit β-CAs from *M. restricta*, *S. cerevisiae*, *C. albicans*, and *C. neoformans*, resulting in growth defects and reduced infectivity [12,31,65,66]. Famotidine, a clinically approved H_2_-receptor antagonist, has also been shown to inhibit fungal β-CA activity [67]. Structural and functional studies on *A. fumigatus* CA (CafA) further support its druggability: high-resolution crystal structures of CafA bound to the potent inhibitor acetazolamide reveal a well-defined active site amenable to structure-guided optimization; moreover, CafA exhibits functional intolerance to nitrate (NO_3_^−^), suggesting that exploiting ion-specific allosteric constraints could yield highly selective antifungals for invasive aspergillosis [32]. Collectively, these findings underscore that CA is not merely a housekeeping enzyme but a bona fide virulence factor whose inhibition compromises pathogen fitness across diverse microbial taxa.

In *M. oryzae*, Dang et al. systematically evaluated the antifungal activity of cordycepin [26]. Phenotypically, cordycepin strongly suppressed colony growth, conidiophore and conidia production, appressorium formation, and overall pathogenicity. Transcriptomic profiling revealed significant downregulation of *MoCA1* and *MoCA5* upon cordycepin treatment. Consistently, Δ*MoCA1* and Δ*MoCA5* mutants exhibited heightened sensitivity to cordycepin compared to the wild type—providing genetic evidence that MoCAs serve as key downstream effectors of cordycepin action. Mechanistically, cordycepin exerts its antifungal effects through coordinated disruption of mitochondrial function and nitrogen metabolism pathways. These findings not only expand our understanding of cordycepin’s mode of action but also identify MoCAs and their associated regulatory networks as critical components of fungal metabolic adaptation to cordycepin-induced stress—highlighting their potential as biomarkers or synergistic targets in antifungal combination therapy.

### 5.2. CO_2_ Capture and Environmental Bioremediation

Carbon dioxide is the primary driver of anthropogenic climate change, prompting global efforts to mitigate emissions and enhance carbon sequestration. CAs have emerged as powerful biocatalysts for CO_2_ capture technologies, owing to their extraordinary catalytic efficiency in accelerating CO_2_ hydration to form bicarbonate (HCO_3_^−^) [68]. To overcome limitations of free enzyme instability and low reusability, researchers have immobilized CAs onto diverse solid supports—including polymeric membranes, nanomaterials, and hollow-fiber reactors—to significantly improve CO_2_ absorption kinetics, operational stability, and scalability [69,70]. For instance, immobilization of CA onto hollow-fiber membranes maximizes gas–liquid interfacial area, thereby enhancing mass transfer and CO_2_ conversion efficiency. Advances in enzyme carrier engineering and immobilization methodologies have enabled the development of continuous, scalable, and environmentally sustainable bioprocesses that integrate high enzymatic turnover with green chemistry principles—paving the way for large-scale conversion of CO_2_ into value-added chemicals (e.g., bicarbonates, carbonates, or bio-based polymers) [71].

Fungal CAs hold exceptional promise in environmental biotechnology, particularly for carbon capture and storage (CCS). The CA-catalyzed conversion of CO_2_ to HCO_3_^−^ is not only essential for supplying inorganic carbon for cellular metabolism but is also evolutionarily conserved across pathogenic fungi—including *C. albicans*, *S. cerevisiae*, and *C. neoformans*—where it serves as a central node in CO_2_ sensing and virulence regulation [72]. Beyond carbon management, engineered fungal CA systems are being harnessed for heavy metal remediation. For example, optimized CA from the marine yeast *Rhodotorula* sp. enhances microbial biomineralization, promoting the co-precipitation and encapsulation of toxic Zn^2+^ and Cr^6+^ ions within vaterite (CaCO_3_) crystals—a robust, eco-friendly strategy to immobilize heavy metals and restrict their mobility in contaminated soils and wastewater [71]. Compared with conventional chemical CO_2_ capture methods—which often require high energy input, extreme temperatures/pressures, and generate hazardous waste—enzyme-based systems operate under mild conditions (ambient temperature, neutral pH), exhibit high specificity and catalytic turnover, and align with principles of green and sustainable chemistry [73].

## 6. Future Directions and Prospects of Fungal CAs

### 6.1. Elucidating the Regulatory Mechanisms of MoCAs in M. oryzae

CAs play essential roles in early fungal development, influencing fatty acid biosynthesis, conidia germination, and hyphal growth [6]. While multiple *CA* genes have been identified across fungal genomes, their specific physiological functions, regulatory mechanisms, and interactions with other metabolic pathways—such as cyanate metabolism or cAMP signaling—remain poorly understood. Further studies using gene knockout, expression analysis, and subcellular localization will be necessary to clarify the functional specificity of each CA isozyme [6,24,25]. In particular, for the MoCA family members in *M. oryzae*, several key knowledge gaps require urgent investigation:

Functional Redundancy and Specificity Analysis: *M. oryzae* harbors at least six CA family members, and functional compensation has been observed in Δ*MoCA6* mutants, where other *MoCAs* are upregulated, suggesting complex compensatory mechanisms within the family [25]. To systematically dissect the functional specificity of each MoCA, future work should focus on generating systematic multi-gene knockout strains (e.g., Δ*MoCA1*/Δ*MoCA5* double knockouts, Δ*MoCA1*/Δ*MoCA2–6* combinations), as well as constructing site-mutant strains (e.g., replacing the zinc-coordinating cysteine residue with serine) to distinguish catalytic from non-catalytic functions.

Dynamic Monitoring During Host Invasion: Current understanding of MoCAs largely stems from in vitro phenotypic analyses, but there is a lack of tools to monitor CA activity and local HCO_3_^−^/pH dynamics during active host invasion. Future research should develop ratio-based pH fluorescent probes [74] for real-time monitoring of subcellular pH changes, FRET-based HCO_3_^−^ sensors [75], activity-based probes (ABPs) for labeling active CA proteins in complex samples, and single-cell transcriptomics to analyze spatial expression patterns of MoCAs in infected tissue sections.

Urgent Need for Structural Biology Studies: In the field of structural biology, the determination of three-dimensional conformations of key enzymes is crucial for elucidating their catalytic mechanisms and facilitating the development of targeted inhibitors. Recent studies have utilized crystallographic analysis to reveal the catalytic mechanism of β-carbonic anhydrase CafB from *A. fumigatus*, providing important theoretical insights for the development of novel inhibitory strategies against this pathogenic fungus [76]. To date, no crystal structure of a plant pathogenic fungal CA has been reported. High-resolution structural determination of CA monomers and their complexes with substrates or inhibitors is one of the most pressing needs in this field, as it will provide critical insights into catalytic mechanisms and guide the rational design of novel antifungal agents.

Mechanistic Study on the Regulation of Intracellular pH Homeostasis by MoAE4-MoCAs: Although the regulatory interaction between MoAE4 and MoCA1 has been well documented [50], our recent findings speculate that MoAE4 transcriptionally regulates all members of the MoCA family in a unidirectional manner, with MoAE4 likely positioned upstream of this regulatory cascade. To gain a comprehensive understanding of pH/HCO_3_^−^ dynamics during *M. oryzae* infection of host plant cells, future investigations should prioritize the construction of *MoAE4-MoCA1* (and *MoAE4-MoCAs*) double-deletion mutants, with the aim of dissecting the functional interplay between MoAE4 and MoCAs. Such efforts will not only unravel the MoAE4-MoCAs axis governing acid–base homeostasis, but also deepen our understanding of the molecular basis of *M. oryzae* pathogenicity, thereby offering new perspectives on the intricate pathogenic mechanisms of this devastating fungal pathogen.

### 6.2. Development of Selective Inhibitors

Due to the high similarity of active site structures among all carbonic anhydrases, achieving subtype-specific inhibition remains challenging. However, significant differences exist between fungal and human CAs in terms of structure, subcellular localization, and regulatory mechanisms, offering a solid foundation for designing highly selective inhibitors with minimal off-target toxicity. Recent advances, combining structural biology with computer-aided drug design, have enabled the development of novel scaffolds for highly selective CA inhibitors, offering a reliable theoretical basis for the development of potential CAII-specific inhibitors [77]. Therefore, the development of targeted fungal CA inhibitors not only holds promise as a new antifungal strategy but also serves as a powerful tool for investigating the pathogenic mechanisms of fungal pathogens [20]. To advance promising inhibitors into preclinical and clinical studies, comprehensive assessments of their safety and efficacy are essential.

In recent years, deep learning generative models have been widely applied in various fields of drug discovery, including but not limited to drug design and development [78]. With continuous advancements in drug design and development technologies, as well as the progress in techniques for protein structure determination, several selective inhibitors targeting CA enzyme isomers have been successfully developed [79]. The core objective of drug discovery lies in designing compounds that can efficiently and specifically bind to target molecules. Most small-molecule drugs act on proteins, and the rational design of heterocyclic CA inhibitors has become a major focus of current research [80,81]. And, artificial intelligence (AI) has made significant progress in elucidating the three-dimensional structures of proteins, greatly enhancing the efficiency and accuracy of drug design [82]. By leveraging AI technologies, researchers are now able to identify potential drug targets more rapidly and optimize the structural and functional properties of candidate compounds. Future research should combine high-resolution structural analysis, AI-driven drug discovery, and high-throughput screening platforms to develop species- or compartment-specific inhibitors targeting specific CA subtypes, such as β-CAS2 or α-CAS4. Additionally, evaluating the impact of these inhibitors on non-pathogenic fungi (e.g., industrial filamentous fungi) will help assess ecological safety and define application boundaries, facilitating their translation into agricultural protection, food preservation, and biomedical applications [83].

### 6.3. Protein Engineering and Enzyme Optimization

Rational design and directed evolution offer powerful strategies to enhance the stability, catalytic activity, and substrate specificity of fungal carbonic anhydrases. Despite their biological importance, natural fungal CAs face limitations in industrial and medical applications due to poor stability under harsh conditions (e.g., high temperature, extreme pH, organic solvents, or high salinity), as well as suboptimal catalytic efficiency and substrate preference.

X-ray free-electron lasers (XFELs) combined with serial femtosecond crystallography have provided a novel approach for investigating the reaction dynamics of enzymes in structural biology. This technique successfully resolved the XFEL structure of carbonic anhydrase II, which was compared with NMR, synchrotron X-ray, and neutron single-crystal structures [84]. Additionally, studies have demonstrated the successful development of an efficient method for screening and predicting isomer-specific inhibitors of carbonic anhydrase through the integration of machine learning, cheminformatics, and experimental validation, while also elucidating the structural determinants underlying inhibitor selectivity [85]. Future progress will rely on structural biology (including high-resolution X-ray crystallography and cryo-electron microscopy) and computational approaches (molecular dynamics simulations, AI-based protein folding prediction) to elucidate catalytic mechanisms, identify key active site residues, and characterize flexible regions in fungal CAs [40,86]. This knowledge will guide targeted engineering efforts to develop stable and customized enzymes for biotechnological and therapeutic applications. Furthermore, exploring new applications of these enzymes in CO_2_ capture, biomineralization, and biosensing will expand their utility in diverse fields.

### 6.4. Expanding Research on CA in Plant Pathogenic Fungi

Currently, functional studies on carbonic anhydrases in plant pathogenic fungi have primarily focused on *M. oryzae*. However, as our understanding of the pathogenic mechanisms of plant pathogenic fungi deepens, it is essential to extend CA research to a broader range of fungal species, including *Botrytis cinerea*, *Verticillium dahliae*, and *Fusarium graminearum*. Systematic analysis of CA family members in these fungi can provide insights into their roles in pathogenicity, adaptation, and life cycle evolution.

Predictive analyses suggest that the number and types of CA family members vary significantly among different plant pathogenic fungi. For instance, *B. cinerea* contains five CA members, comprising one α-CA and four β-CAs; *V. dahliae* has six CA members, with three α-CAs and three β-CAs; and *F. graminearum* possesses seven CA members, including three α-CAs and four β-CAs. Despite these differences in the number and classification of CA members, all identified CAs exhibit Zn^2+^-dependent CO_2_ hydration activity, indicating a conserved functional basis in metabolic regulation across these species.

Based on these observations, we propose that carbonic anhydrases (CAs) may play critical roles in various physiological and pathogenic processes of plant pathogenic fungi. Systematic comparative analysis of the evolutionary characteristics of CA families across different plant pathogenic fungi could help reveal the mechanisms underlying gene expansion and contraction of the CA family during the adaptive evolution of fungal pathogens. However, whether the zinc-coordinating residues and active site structures of fungal CAs are sufficiently conserved to support the design of specific inhibitors remains an issue warranting further investigation.

Elucidating the specific functions of CAs in the pathogenic processes of fungal pathogens not only enhances our understanding of fungal pathogenesis but also provides a theoretical basis and molecular target for the development of broad-spectrum antifungal strategies. By comprehensively analyzing and comparing the mechanisms of CAs in human and plant pathogenic fungi, it is possible to identify their conserved features as well as differences arising from system-specific adaptations. In addition, advanced molecular techniques such as CRISPR-Cas9 gene editing and subcellular localization can be employed to investigate the functional roles of CAs in plant pathogenic fungi, thereby offering experimental insights for the development of antifungal agents targeting CAs and for disease control strategies.

In conclusion, expanding CA research to a wider range of plant pathogenic fungi, combined with systematic evolutionary analysis and functional validation, will offer important scientific insights into fungal pathogenic mechanisms and contribute to the design of novel antifungal agents.

## 7. Conclusions

Fungal carbonic anhydrases (CAs) represent a class of crucial zinc-containing metalloenzymes that play pivotal roles in the physiological metabolism, environmental adaptation, and pathogenicity of fungi. This review systematically summarizes the classification diversity, structural characteristics, and subcellular localization of fungal CAs. Focusing on *M. oryzae* as a model organism, this work integrates recent advances in the field, highlighting the significant evolutionary diversity of CAs across different species. Variations in gene number, type, and subcellular localization reflect adaptive strategies to diverse ecological environments.

In *M. oryzae*, five CA members are localized to the mitochondria, forming a unique mitochondrial CA functional network. These enzymes collectively participate in key biological processes such as CO_2_ sensing and signaling, conidia development, metabolic substrate supply, and virulence expression. Recent studies have further revealed functional interactions among five CA members, demonstrating their involvement in nitrogen metabolism, ATP synthesis, and pathogenicity regulation.

Due to the structural differences between fungal and human CAs, as well as the central role of CAs in the pathogenicity of fungal pathogens, these enzymes have emerged as promising targets for the development of novel antifungal agents. Compounds such as cordycepin have been shown to effectively inhibit CA activity in *M. oryzae*, demonstrating considerable application potential. Moreover, fungal CAs show great promise in environmental biotechnology applications, including CO_2_ capture, biomineralization, and heavy metal immobilization.

Despite challenges such as the development of selective inhibitors and the limited stability of the enzymes, ongoing advancements in structural biology, artificial intelligence-driven drug discovery, and protein engineering are gradually addressing these issues. In the future, integrating in vitro experiments, computational modeling, and in vivo studies will further advance both fundamental and applied research on fungal CAs. This will provide innovative strategies and solutions for the green control of plant pathogenic fungi, new antifungal therapies, and sustainable environmental development.

## Figures and Tables

**Table 1:** Subcellular localization and functional characterization of carbonic anhydrases in fungi.

Fungal Species	CA Name	Subcellular Localization	Functional Characteristics
*Aspergillus oryzae*	AoCA	Predicted secreted/extracellular	CO_2_ hydration; Catalytic activity; Imidazole-activated.
*Sordaria macrospora*	CAS4	Secreted/extracellular	HCO_3_^−^ production; Vegetative growth; ascospore germination.
*Paracoccidioides*	CA4	Predicted secreted/extracellular	Fungal pathogenicity; Fatty acid biosynthesis.
*Candida albicans*	Nce103p	Cell wall and plasma membrane (main); cytoplasm and mitochondria (partial)	CO_2_ sensing; HCO_3_^−^ homeostasis; cAMP-PKA signaling; growth and virulence.
*Candida parapsilosis*	Nce103p	Cell wall and plasma membrane (main); cytoplasm and mitochondria (partial)	CO_2_ sensing; HCO_3_^−^ regulation; environmental adaptation.
*Sordaria macrospora*	CAS1	Cytoplasm	Fruiting body and ascospore germination; Overlaps with CAS2, yet diverges.
*Sordaria macrospora*	CAS2	Mitochondria	Fruiting body and ascospore germination; Essential for hyphal growth and conidial germination.
*Sordaria macrospora*	CAS3	Cytoplasm	Fruiting body and ascospore germination; Overlaps with CAS2, yet diverges.
*Aspergillus fumigatus*	CafA	Mitochondria	Essential for growth under ambient air; Affects conidial yield and participates in the regulation of conidial development.
*Aspergillus fumigatus*	CafB	Cytoplasm	Essential for growth under ambient air.
*Aspergillus fumigatus*	CafC	Cytoplasm	Affects conidial yield; Participates in the regulation of conidial development.
*Aspergillus fumigatus*	CafD	Mitochondria	Unknown physiological function.
*Magnaporthe oryzae*	MoCAs	Mitochondria (Excluding MoCA3)	Conidial development; Germination; Appressorium formation; Pathogenicity; Affect ATP synthase; Involved in nitrogen metabolism(Excluding MoCA3).
*Cryptococcus neoformans*	Can1	Not determined	CO_2_ sensing and virulence regulation during infection of human hosts.
*Cryptococcus neoformans*	Can2	Not determined	Essential for growth under ambient air; CO_2_ sensing and signal transduction; cAMP-PKA signaling pathway; Sexual reproduction and conidial formation; Virulence and pathogenicity; Fatty acid synthesis.
*Aspergillus nidulans*	CanA	Cytoplasm	Participates in the regulation of conidial development.
*Aspergillus nidulans*	CanB	Cytoplasm	Essential for growth under ambient air.

## Data Availability

The original contributions presented in the study are included in the article, further inquiries can be directed to the corresponding author.

## Nomenclature

The following compact nomenclatures are used in this manuscript:

Terminology	Definition
Conidia	The primary infectious propagule produced during the asexual reproductive stage, consists of three-celled pyriform conidia.
Germination	Germination refers to the transition of conidia from a dormant state to polarized growth, characterized by germ tube emergence, followed by appressorium formation during early infection.
Appressorium	A unicellular, spherical specialized infection structure differentiated from the tip of the germ tube arising from a germinating conidium.
Penetration Peg	A minute, tube- or peg-shaped infection structure formed at the appressorial pore, propelled by the high hydrostatic pressure generated within the mature appressorium, which enables direct physical rupture of the host cuticle and underlying epidermal cell wall.
Turgor Pressure	The mature appressorium accumulates high concentrations of glycerol and polyols to generate an osmotic turgor pressure of up to 8.0 MPa. This pressure, confined by the melanized cell wall, drives the penetration peg to breach the host cuticle and underlying cell wall.
Melanin	An irregular, light-absorbing macromolecular polymer that serves as a dark pigment in the appressorial cell wall, conferring mechanical rigidity crucial for resisting high turgor pressure and contributing significantly to the organism’s self-protection.
Invasive Hyphae	Invasive hyphae are specialized intracellular fungal hyphae that develop after host penetration and colonize rice cells before spreading to adjacent cells.
Biotrophic Stage	A distinct developmental stage in which invasive hyphae, after establishing a parasitic association inside living host cells, derive nutrients from viable host cells to sustain their expansion and colonization, while simultaneously repressing host immune responses.
Reactive Oxygen Species (ROS)	A group of highly reactive oxygen species catalytically produced by fungal endogenous enzymes, most notably NADPH oxidases, throughout the infection-associated morphogenetic program of the pathogen.
Mitochondrial Matrix	The mitochondrial matrix, enclosed by the inner membrane, contains mtDNA, ribosomes, and diverse metabolic enzymes and intermediates, and is the central hub for the TCA cycle and ATP synthesis.
cAMP-PKA Signaling Pathway	A conserved signal transduction cascade activated by bicarbonate (HCO_3_^−^) via adenylyl cyclase, regulating fungal development, mating, and virulence.
Mitochondrial Respiratory Chain	The mitochondrial respiratory chain is an inner-membrane electron transport system that supports ATP production and mitochondrial energy metabolism.
Complex I	Designated NADH:ubiquinone oxidoreductase, this complex constitutes the largest and entry-level multi-subunit enzyme complex of the mitochondrial respiratory chain. It couples the oxidation of NADH and reduction of ubiquinone with proton translocation across the inner membrane, thereby functioning as a critical rate-limiting step in the generation of proton motive force and the subsequent synthesis of ATP.
Carboxylation Reactions	Carboxylation reactions are HCO_3_^−^-dependent enzymatic reactions that incorporate carbon dioxide/bicarbonate-derived carbon into metabolic substrates, thereby supporting key biosynthetic and anaplerotic pathways.
Acetazolamide (Ace)	Acetazolamide is a small heterocyclic sulfonamide compound known to bind with high affinity to various carbonic anhydrases, acting as a potent carbonic anhydrase inhibitor.
Cordycepin	Cordycepin (3'-deoxyadenosine) is a nucleoside antibiotic consisting of an adenine base linked to a branched-chain deoxypentose sugar. It belongs to the class of nucleoside analogues and is recognized for its diverse bioactive properties.
